# Genetic response to human‐induced habitat changes in the marine environment: A century of evolution of European sprat in Landvikvannet, Norway

**DOI:** 10.1002/ece3.7160

**Published:** 2021-01-18

**Authors:** María Quintela, Àlex Richter‐Boix, Dorte Bekkevold, Cecilie Kvamme, Florian Berg, Eeva Jansson, Geir Dahle, François Besnier, Richard D. M. Nash, Kevin A. Glover

**Affiliations:** ^1^ Institute of Marine Research Bergen Norway; ^2^ CREAF Campus de Bellaterra Autonomous University of Barcelona Barcelona Spain; ^3^ DTU‐Aqua National Institute of Aquatic Resources Technical University of Denmark Silkeborg Denmark; ^4^ Centre for Environment Fisheries and Aquaculture Science (Cefas) Lowestoft UK; ^5^ Institute of Biology University of Bergen Bergen Norway

**Keywords:** evolutionary response, habitat alteration, local adaptation, SNP, sprat

## Abstract

Habitat changes represent one of the five most pervasive threats to biodiversity. However, anthropogenic activities also have the capacity to create novel niche spaces to which species respond differently. In 1880, one such habitat alterations occurred in Landvikvannet, a freshwater lake on the Norwegian coast of Skagerrak, which became brackish after being artificially connected to the sea. This lake is now home to the European sprat, a pelagic marine fish that managed to develop a self‐recruiting population in barely few decades. Landvikvannet sprat proved to be genetically isolated from the three main populations described for this species; that is, Norwegian fjords, Baltic Sea, and the combination of North Sea, Kattegat, and Skagerrak. This distinctness was depicted by an accuracy self‐assignment of 89% and a highly significant *F*_ST_ between the lake sprat and each of the remaining samples (average of ≈0.105). The correlation between genetic and environmental variation indicated that salinity could be an important environmental driver of selection (3.3% of the 91 SNPs showed strong associations). Likewise, Isolation by Environment was detected for salinity, although not for temperature, in samples not adhering to an Isolation by Distance pattern. Neighbor‐joining tree analysis suggested that the source of the lake sprat is in the Norwegian fjords, rather than in the Baltic Sea despite a similar salinity profile. Strongly drifted allele frequencies and lower genetic diversity in Landvikvannet compared with the Norwegian fjords concur with a founder effect potentially associated with local adaptation to low salinity. Genetic differentiation (*F*_ST_) between marine and brackish sprat is larger in the comparison Norway‐Landvikvannet than in Norway‐Baltic, which suggests that the observed divergence was achieved in Landvikvannet in some 65 generations, that is, 132 years, rather than gradually over thousands of years (the age of the Baltic Sea), thus highlighting the pace at which human‐driven evolution can happen.

## Graphical abstract

Anthropogenic activities also have the capacity to create novel niche spaces to which species respond differently. In 1880, Landvikvannet, a freshwater lake on the Norwegian coast of Skagerrak became brackish after being artificially connected to the sea. Today, this lake is home to a population of European sprat, genetically isolated from the three main populations described for this species.

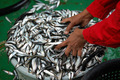

## 1 INTRODUCTION

Humans have dramatically impacted the Earth's surface and promoted striking ecosystem and biodiversity alterations over the course of the last two centuries, hence becoming an evolutionary force of extraordinary influence (Albuquerque et al., 2018; Ceballos et al., 2015; Hooper et al., 2012). Human activities generate major pressures on habitats and organisms and are associated with evolutionary changes that can occur within tens of years, a phenomenon known as “contemporary evolution” (Besnier et al., 2014; Otto, 2018; Pelletier & Coltman, 2018; Stockwell et al., 2003). Human‐driven evolution can happen at a pace and extent that is significantly higher than that of natural causes (Bull & Maron, 2016; Hendry et al., 2008; Palumbi, 2001; Therkildsen et al., 2019). Anthropogenic activities have altered and created novel niche spaces and species' responses to ecosystem alterations vary from avoidance to adaptation, including exploitation (Bull & Maron, 2016).

**FIGURE 1:**
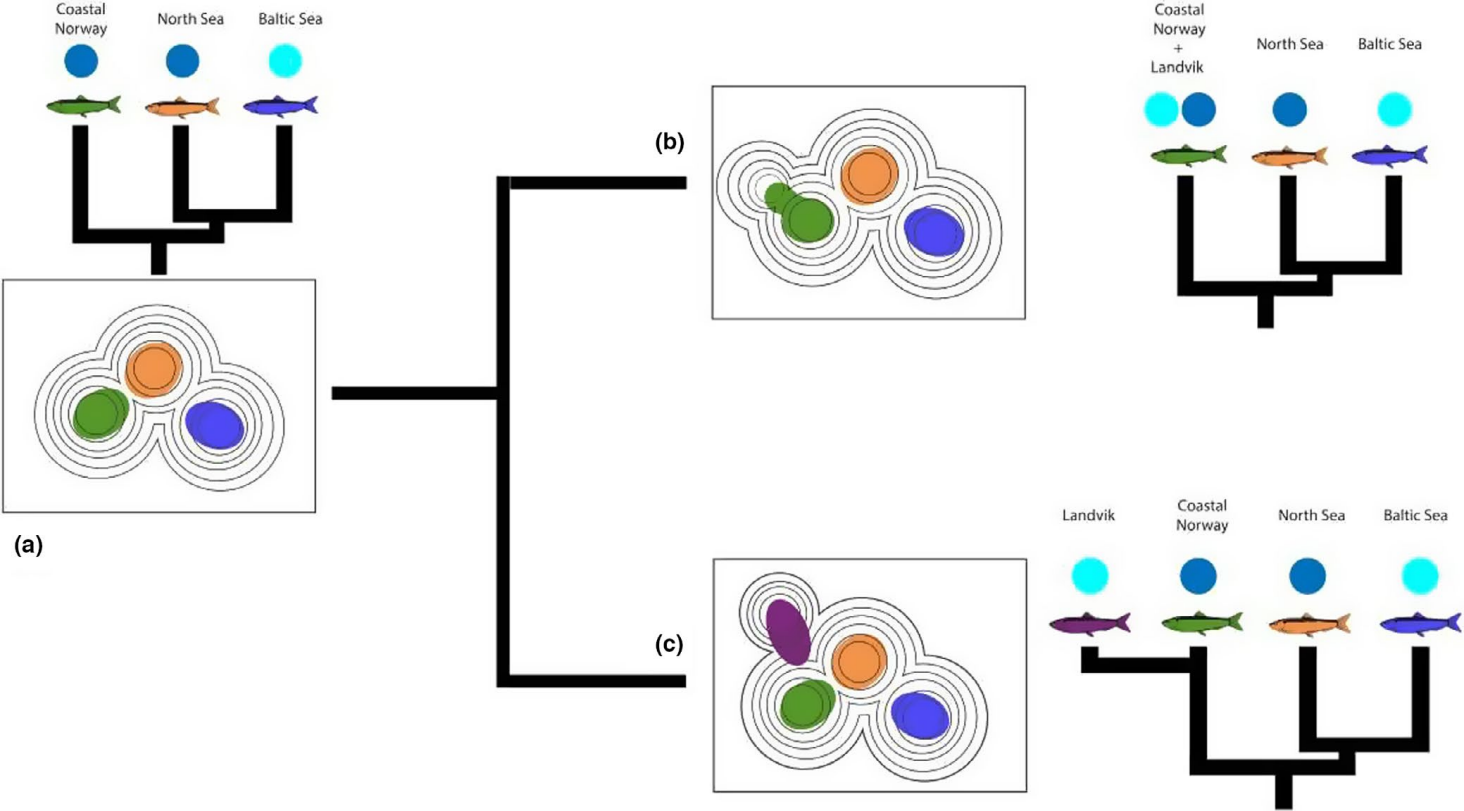
Hypothetical adaptive landscapes showing mean population fitness (color contours) and its genetic consequences (a). Black circles show potential distribution of phenotype/genotype. The starting adaptive landscape has three fitness peaks that are each occupied by its own genetic and adapted population (Coastal Norway, North Sea, and Baltic Sea), where the blue circle of each population depicts the environment (dark blue for marine environment, light blue for brackish environment). When humans' actions created the connection of Landvikvannet with the sea, added a new peak (brackish environment) to the original sprat adaptive landscape. Two plausible scenarios are possible following the creation of the new ecological niche. (b) The neighboring population colonizes the new habitat, but the selection is not enough for the new population to differentiate itself from the ancestral one. (c) Selection in the new environment is strong enough so that the population of the new habitat differs from the ancestral population. In that case, a phenomenon of parallel adaptation to brackish environments can occur

Humans are fundamentally changing connections within and among ecosystems over a wide range of spatial scales and habitat types, hence modifying the levels of connectivity (Crook et al., 2015). Such changes can pose direct threats to communities, but may also create novel environments that influence the evolutionary trajectories of populations and species (Allendorf et al., 2012), and can alter the phenotypic landscapes of species by decreasing or increasing genetic diversity (Figure 1) (Hendry et al., 2017). Many examples of contemporary evolution are associated with colonization events, species introductions, or invasions (Colautti & Lau, 2015; Johnston & Selander, 1964; Reznick & Ghalambor, 2001). Populations colonizing new environmental conditions can be exposed to novel selective forces that lead to adaptive divergence and differentiation from the original population (Björklund & Gustafsson, 2015; Hendry et al., 2002).

The construction of navigation canals is an example of human‐facilitated connectivity between two previously isolated ecosystems (Galil et al., 2007). Canals can link marine and freshwater bodies allowing aquatic organisms to disperse to new areas and eventually colonize novel environments (Crook et al., 2015). One such connectivity change took place in 1880, when the lake Landvikvannet (henceforth denoted as Landvik for abbreviation), on the southern Norwegian Skagerrak coast, was artificially connected to the adjacent marine fjord (Strandfjorden, Grimstad, Norway) by a 3 km long narrow canal (Reddal Canal). The construction of the canal, built to transport logs down to the dockyards by the sea as well as to drain the lake to increase the surface of arable land, lowered the water level in the lake by 3 m, turning the lake brackish as saltwater inflows over the tidal cycle while there is a continuous flux of freshwater from streams into the lake (Kanalkontoret, 1883). This human alteration drove changes in species assemblages, facilitating the colonization of marine species like the Atlantic herring (*Clupea harengus*) (Linnaeus, 1758) and European sprat, *Sprattus sprattus* (Linnaeus, 1758). Although it is unsure when these marine species colonized Landvik, the first sprat sample taken by the Institute of Marine Research dates back to 1999.

The European sprat is a small pelagic fish that is widely distributed from northern Norway to Morocco, the Baltic Sea, the northern Mediterranean basins, and the Black Sea (Debes et al., 2008). Three geographically distinct genetic groups have been described with nuclear markers: (a) Norwegian fjords, (b) Baltic Sea, and (c) a wide‐ranging component spanning the North Sea, Kattegat–Skagerrak in north to the Celtic Sea, and Bay of Biscay in south (Glover et al., 2011; Limborg, Hanel, et al., 2012; Limborg et al., 2009; Quintela et al., 2020). Furthermore, mitochondrial control region revealed two additional demes in the Mediterranean Sea, Gulf of Lyon, and Adriatic Sea (Debes et al., 2008). Differences found in candidate loci for divergent selection between the fresh‐ to brackish water Baltic Sea and fully marine populations suggest that local adaptation to low salinity is likely (Quintela et al., 2020), as has been shown in other Clupeid species such as the Atlantic herring in the Baltic Sea (Guo et al., 2016; Limborg, Helyar, et al., 2012), and the European anchovy (*Engraulis encrasicolus* Linnaeus, 1758) in the Adriatic (Ruggeri et al., 2016) and Tyrrhenian Seas (Catanese et al., 2017). The colonization of Landvik's brackish waters might have been possible due to the sprat's standing genetic variation allowing adaptation to a range of salinities, as conditions in Landvik partly resemble those in the Baltic Sea, the largest brackish water body in the world (Florian Berg, 2018).

The relatively recent colonization of Landvik by sprat provides an opportunity to study a contemporary evolution process, testing whether the creation of this new environment has promoted genetic differentiation from standing variation through ecological adaptation. This happens when barriers to gene flow evolve between populations due to divergent selection, with niche adaptation and competition as driving mechanisms (Bolnick, 2004; Schluter, 2000). Landvik's salinity is similar to that of parts of the Baltic Sea, which thus allows the use of it as a replicate model to study parallel evolution and the role of the environment in ecologically driven speciation (Bailey et al., 2017; Bolnick et al., 2018).

To test for local adaptation and parallel evolution, we first characterized Landvik sprat with a suite of recently developed SNP markers and investigated the origin and connectivity of the lake population using a set of 42 geographically explicit samples, most of which were described in Quintela et al. (2020). Secondly, we investigated whether loci putatively under selection could be identified across these samples. Correlation between outlier loci and two environmental variables, salinity and temperature, was examined to test the potential role of selection in population divergence, and the possibility to identify genetic signals of parallel evolutionary change between Landvik and the Baltic Sea populations with respect to the marine populations.

## 2 MATERIALS AND METHODS

### 2.1 Sampling and environmental data

**FIGURE 2:**
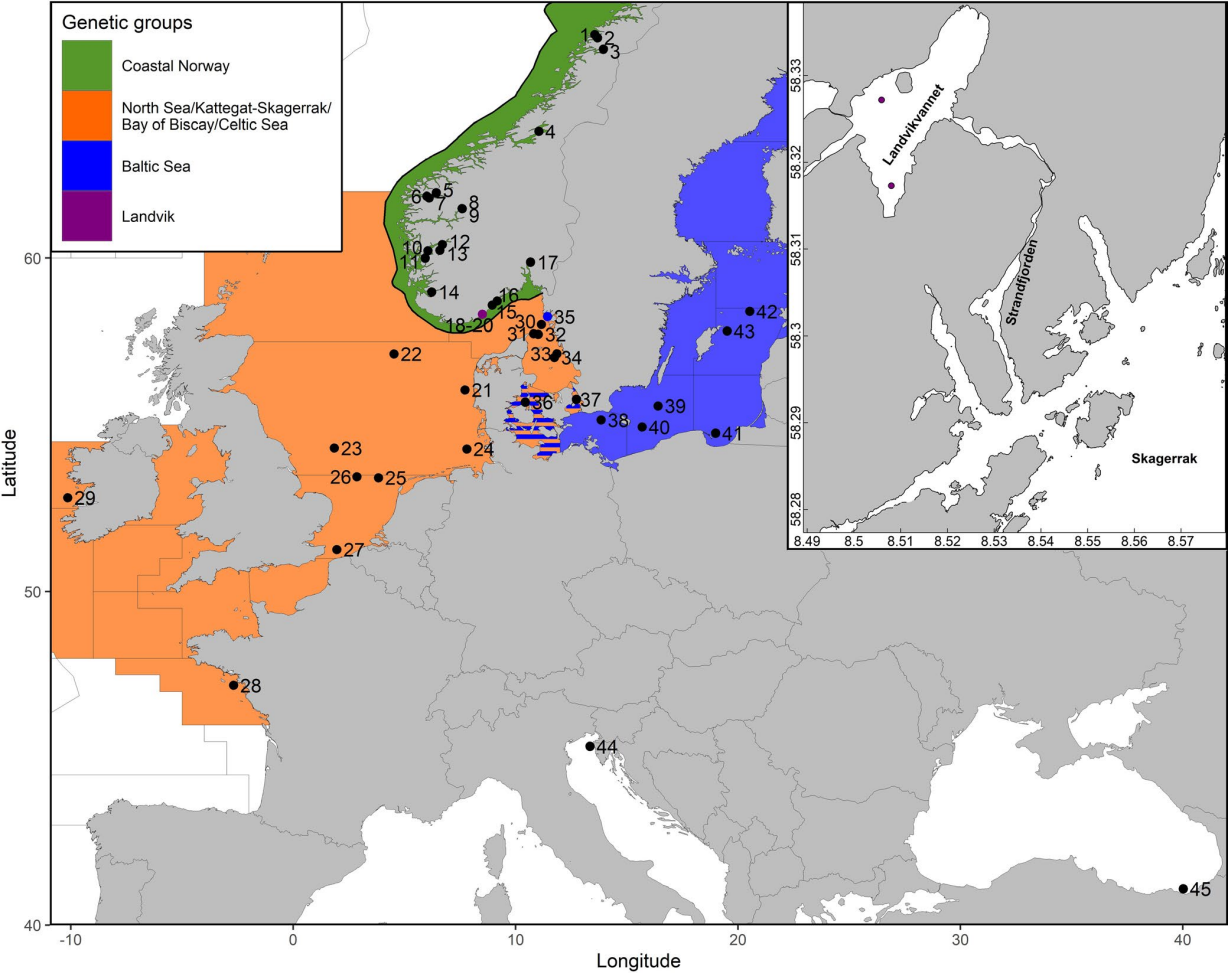
Map of the sampling sites as well as detailed view of Landvikvannet. Codes and associated full names of sampling locations can be found in Table 1. The colors depict the genetic clusters according to STRUCTURE. Landvikvannet samples are coded as 18–20

Three samples of sprat from Landvik, comprising a total of 300 individuals, were collected in 2012, 2015, and 2019, respectively. In addition, to compare among local populations in the area, a further 79 (immature juvenile) individuals were collected in 2019 in two Norwegian fjords in the vicinity of Landvik (Tvedestrandsfjord and Sørfjord) from a beach seine survey. These five samples were analyzed and compared with genotype data from 40 reference samples of sprat, 2,425 individuals in total, collected from a range of locations in the Atlantic and the Baltic Sea, as well as in the Adriatic and Black Seas, representing southern outgroups (Table 1, Figure 2). Genetic structure in the 40 reference samples was determined in Quintela et al. (2020), showing three highly distinct and relatively homogenous groups: (a) Norwegian fjords; (b) Baltic Sea; and (c) Northeast Atlantic including the North Sea, Kattegat–Skagerrak, Celtic Sea and Bay of Biscay. Evidence of genetic admixture and possibly physical mixing was detected in the transition zone between the North and Baltic seas, but not elsewhere.

**TABLE 1:** Summary statistics of sprat genotyped at 91 SNP: Sampling site within geographic regions, time of collection (year/month), coordinates, number of individuals; observed heterozygosity, *H_o_* (mean ± *SE*); unbiased expected heterozygosity, *uH_e_* (mean ± *SE*); inbreeding coefficient, *F*_IS_ (mean ± *SE*); number of deviations from Hardy–Weinberg equilibrium (HWE) at *α* = 0.05; number of deviations from Linkage Disequilibrium (LD) at *α* = 0.05 both before and after (B) Bonferroni correction

Region	No	Site	Year	Month	Code	Latitude	Longitude	No ind	*H_o_*	*uH_e_*	*F*_IS_	HWE (B)	LD (B)	Temp	Salin
Norwegian fjords	1	Holandsfjord	2008	12	HOL	66,705	13,562	31	0.278 ± 0.018	0.282 ± 0.016	0.003 ± 0.021	7 (0)	125 (1)	10.15	32.98
	2	Melfjord	2008	12	MEL	66,610	13,686	79	0.286 ± 0.016	0.281 ± 0.015	−0.021 ± 0.013	7 (0)	193 (2)	12.06	32.62
	3	Finneidfjord	2008	12	FIN	66,268	13,942	75	0.276 ± 0.015	0.286 ± 0.015	0.016 ± 0.015	8 (0)	178 (2)	12.17	33.05
	4	Stjørdalsfjord	2008	12	TRH	63,807	11,041	80	0.281 ± 0.015	0.286 ± 0.015	0.005 ± 0.013	5 (1)	193 (4)	12.21	32.20
	5	Nordfjord	2015	12	NOR1	61,959	6,429	39	0.275 ± 0.017	0.286 ± 0.016	0.024 ± 0.020	7 (2)	141 (2)	12.27	32.98
	6	Nordfjord	**2001**	**5**	NOR2	61,860	6,004	74	0.284 ± 0.017	0.286 ± 0.016	0.008 ± 0.014	7 (0)	196 (3)	12.19	32.36
	7	Nordfjord	2015	12	NOR3	61,811	6,111	49	0.264 ± 0.016	0.283 ± 0.016	0.053 ± 0.019	7 (3)	153 (2)	12.19	32.36
	8	Sognefjord	2008	11	SOG1	61,489	7,589	47	0.264 ± 0.016	0.278 ± 0.016	0.024 ± 0.018	10 (1)	155 (2)	12.47	31.99
	9	Sognefjord	2015	12	SOG2	61,485	7,597	116	0.266 ± 0.014	0.283 ± 0.015	0.047 ± 0.014	16 (2)	202 (4)	12.47	31.99
	10	Hardangerfjord	2015	12	HAR1	60,225	6,058	100	0.269 ± 0.014	0.283 ± 0.015	0.032 ± 0.010	7 (0)	203 (3)	12.87	33.09
	11	Hardangerfjord	2008	11	HAR2	60,000	5,935	77	0.269 ± 0.015	0.284 ± 0.016	0.035 ± 0.016	12 (2)	172 (3)	13.78	31.74
	12	Hardangerfjord	2008	11	HAR3	60,410	6,701	46	0.286 ± 0.016	0.284 ± 0.015	−0.018 ± 0.017	5 (0)	160 (2)	12.87	31.64
	13	Hardangerfjord	2008	11	HAR4	60,236	6,589	99	0.278 ± 0.015	0.282 ± 0.015	0.004 ± 0.013	9 (3)	188 (2)	12.87	33.09
	14	Lysefjord	2008	11	LYS	58,987	6,221	100	0.273 ± 0.015	0.286 ± 0.016	0.032 ± 0.013	7 (1)	175 (4)	11.07	31.75
	15	Tvedestrandsfjord	2019	9	TVE	58,589	8,982	37	0.251 ± 0.018	0.267 ± 0.018	0.033 ± 0.022	11 (1)	129 (1)	13.68	30.17
	16	Sørfjord	2019	9	SORF	58,730	9,079	42	0.263 ± 0.019	0.264 ± 0.017	−0.007 ± 0.018	5 (1)	142 (1)	13.68	31.44
	17	Oslofjord	2007	9	OSL	59,881	10,665	89	0.269 ± 0.016	0.281 ± 0.016	0.034 ± 0.013	7 (1)	207 (2)	13.68	31.00
Landvik	18	Landvikvannet	2012	5	LAND12	58,317	8,491	27	0.239 ± 0.022	0.231 ± 0.019	−0.039 ± 0.020	3 (0)	112 (1)	14.86	16.00
	19	Landvikvannet	2015	3	LAND15	58,317	8,491	205	0.225 ± 0.017	0.234 ± 0.018	0.025 ± 0.012	11 (4)	186 (11)	14.86	16.00
	20	Landvikvannet	2019	9	LAND19	58,317	8,491	68	0.251 ± 0.017	0.264 ± 0.017	0.031 ± 0.018	11 (1)	194 (1)	14.86	16.00
North Sea,Kattegat–Skagerrak	21	North Sea	2018	7	NS1	56,049	7,723	57	0.214 ± 0.018	0.231 ± 0.018	0.082 ± 0.024	5 (1)	155 (1)	14.33	32.94
	22	North Sea	**2015**	**5**	NS2	57,130	4,526	77	0.231 ± 0.018	0.238 ± 0.018	0.008 ± 0.013	6 (1)	136 (1)	15.55	34.44
	23	North Sea	2008	?	NS3	54,307	1,842	93	0.233 ± 0.018	0.246 ± 0.018	0.029 ± 0.014	5 (1)	138 (1)	14.11	34.61
	24	North Sea	**2005**	**5**	NS4	54,277	7,802	59	0.224 ± 0.019	0.235 ± 0.019	0.042 ± 0.018	16 (3)	127 (1)	16.07	31.43
	25	North Sea	2016	8	NS5	53,417	3,833	40	0.229 ± 0.019	0.234 ± 0.019	0.027 ± 0.020	3 (1)	127 (1)	16.65	34.48
	26	North Sea	2016	8	NS6	53,449	2,858	38	0.231 ± 0.018	0.241 ± 0.019	0.014 ± 0.017	9 (2)	100 (1)	14.34	34.11
	27	English Channel	**2009**	**6**	EC	51,265	1,959	50	0.218 ± 0.018	0.228 ± 0.019	0.019 ± 0.016	6 (1)	109 (1)	15.67	34.38
	28	Bay of Biscay	2008	8	BoB	47,192	−1,318	57	0.214 ± 0.018	0.234 ± 0.019	0.085 ± 0.023	4 (0)	111 (1)	14.52	35.04
	29	Celtic Sea	2009	10	CEL	52,814	−9,856	79	0.242 ± 0.018	0.245 ± 0.019	−0.007 ± 0.013	5 (1)	121 (1)	13.58	34.16
	30	Kattegat	2018	6	SK1	58,011	11,156	58	0.231 ± 0.019	0.239 ± 0.019	0.017 ± 0.016	3 (1)	114 (1)	15.79	30.65
	31	Kattegat	**2006**	**3**	SK2	57,734	10,809	38	0.227 ± 0.020	0.227 ± 0.019	−0.004 ± 0.019	9 (2)	81 (1)	15.26	32.37
	32	Kattegat	2018	9	SK3	57,718	11,015	38	0.211 ± 0.018	0.239 ± 0.019	0.107 ± 0.026	9 (2)	140 (1)	15.33	31.06
	33	Kattegat	2018	7	SK4	57,134	11,854	41	0.218 ± 0.018	0.230 ± 0.019	0.021 ± 0.018	7 (2)	100 (1)	16.32	30.65
	34	Kattegat	2018	7	SK5	57,021	11,744	73	0.228 ± 0.019	0.241 ± 0.019	0.044 ± 0.018	4 (1)	125 (1)	15.62	22.65
	35	Uddevalla fjord	**2008**	**5**	UV	58,250	11,428	59	0.232 ± 0.017	0.244 ± 0.018	0.023 ± 0.016	11 (5)	133 (1)	16.05	29.10
	36	Great Belt	**2006**	**3**	GB	55,684	10,437	47	0.251 ± 0.018	0.254 ± 0.017	0.005 ± 0.016	7 (1)	137 (1)	14.56	23.64
	37	Øresund	**2006**	**3**	ØS	55,767	12,731	46	0.263 ± 0.019	0.257 ± 0.018	−0.025 ± 0.017	12 (4)	131 (1)	14.83	17.38
Baltic Sea	38	Arkona Basin	**2006**	**5**	AB	55,147	13,845	59	0.232 ± 0.018	0.237 ± 0.019	0.006 ± 0.015	5 (1)	127 (1)	12.81	7.96
	39	Bornholm Basin N	**2006**	**3**	BBN	55,571	16,408	39	0.224 ± 0.020	0.226 ± 0.019	−0.016 ± 0.016	4 (0)	103 (1)	14.16	7.37
	40	Bornholm Basin S	**2006**	**3**	BBS	54,933	15,690	43	0.238 ± 0.020	0.238 ± 0.019	−0.014 ± 0.017	6 (0)	88 (1)	17.32	7.75
	41	Gdansk Deep	**2006**	**3**	GD	54,751	18,993	56	0.227 ± 0.019	0.238 ± 0.019	0.036 ± 0.019	6 (2)	121 (1)	16.39	7.54
	42	Gotland Basin	**2006**	**5**	GOTB	58,406	20,526	55	0.225 ± 0.019	0.232 ± 0.019	0.007 ± 0.015	5 (0)	130 (1)	13.56	6.94
	43	Baltic Sea, Gotland	**2006**	**3**	GOT	57,815	19,509	56	0.240 ± 0.022	0.223 ± 0.019	−0.053 ± 0.017	8 (0)	110 (1)	15.57	6.94
Out	44	Adriatic Sea	2005	12	ASA	45,360	13,340	45	0.179 ± 0.018	0.195 ± 0.020	0.050 ± 0.021	8 (3)	75 (0)	na	na
	45	Black Sea	2008	12	BS	41,087	40,027	21	0.193 ± 0.020	0.208 ± 0.020	0.036 ± 0.027	9 (0)	47 (0)	na	na

Note

Samples from ripe individuals are depicted in boldface type in the year/month columns. Temperature (°C) and salinity were measured at 10 m depth (summer values averaged from 2005–2012).

Spawners and embryos have been identified as the most temperature‐sensitive stages in the life cycle of fish (Dahlke et al., 2020). Data about temperature and salinity corresponding to the average summer values for the period 2005–2012 were retrieved from NOAA database (National Oceanic and Atmospheric Administration). The depth at which measurements were chosen was 10 m for being relevant both for spawners and embryos (Table 1).

### 2.2 DNA isolation and genotyping

DNA was extracted from fin clips stored in ethanol using the Qiagen DNeasy 96 Blood & Tissue Kit in 96‐well plates, each of which contained two or more negative controls. All 45 samples were genotyped with the 91 SNPs for which protocols are described in their entirety in Quintela et al. (2020). In addition, a subset of 15 of the 45 samples was genotyped with eight microsatellite markers (see Table A1), as described in Glover et al. (2011). The main aim of the second set was to estimate genetic diversity through allelic richness, and hence, results derived of the microsatellite data will be mainly presented in Appendix 1.

### 2.3 Statistical analysis

All statistical analyses were performed separately for SNPs and microsatellites. The observed (*H_o_*) and unbiased expected heterozygosity (*uH_e_*) as well as the inbreeding coefficient (*F*_IS_) were computed for each sample with GenAlEx v6.1 (Peakall & Smouse, 2006). The genotype frequency of each locus and its direction (heterozygote deficit or excess) was compared with Hardy–Weinberg expectations (HWE) using the program GENEPOP 7 (Rousset, 2008), as was linkage disequilibrium (LD) between pairwise loci.

Landvik sprat were compared with the remaining collections using pairwise *F*_ST_ (Weir & Cockerham, 1984) computed with ARLEQUIN v.3.5.1.2 (Excoffier et al., 2005). The Bayesian clustering approach implemented in STRUCTURE v. 2.3.4 (Pritchard et al., 2000), and conducted using the software ParallelStructure (Besnier & Glover, 2013), was used to identify genetic groups under a model assuming admixture and correlated allele frequencies without using population information as a prior. Ten runs with a burn‐in period consisting of 100,000 replications and a run length of 1,000,000 MCMC iterations were performed for *K* = 1 to *K* = 7 clusters. To determine the number of genetic groups, STRUCTURE output was analyzed using two approaches: (a) the ad hoc summary statistic Δ*K* of Evanno et al. (2005), and (b) the four statistics (MedMed, MedMean, MaxMed, and MaxMean) both implemented in StructureSelector (Li & Liu, 2018). The ten runs for the selected Ks were then averaged with CLUMPP v.1.1.1 (Jakobsson & Rosenberg, 2007) using the FullSearch algorithm and the G′ pairwise matrix similarity statistic, and graphically displayed using barplots. Furthermore, the relationships between Landvik and the reference samples were examined using discriminant analysis of principal components, DAPC (Jombart et al., 2010) implemented in *adegenet* (Jombart, 2008), as well as with the principal coordinates analysis (PCoA) built using Nei (1978)'s genetic distance between pairs of populations with GenAlEx v6.1 (Peakall & Smouse, 2006). To examine demographic relationships between geographically explicit samples, the genetic distance, measured as *F*_ST_/(1 − *F*_ST_), between the northernmost sample (HOL) and all other samples (excluding the southern European outgroups) was plotted against the corresponding shortest waterway distance, calculated using the path function in GoogleEarth. The assignment of individuals to STRUCTURE genetic clusters was conducted with the program GeneClass 2 (Piry et al., 2004) using the Rannala and Mountain (1997) method of computation. Finally, a neighbor‐joining (NJ) tree based upon pairwise Nei's genetic distance D_A_ (Nei et al., 1983) for all SNPs was constructed with the software POPTREE2 (Takezaki et al., 2010) using 1,000 bootstraps and visualized using FigTree 1.4.3 (Rambaut, 2009).

Two analytic approaches, BayeScan (Foll & Gaggiotti, 2008) and LOSITAN (Antao et al., 2008), were combined to detect loci deviating from neutral expectations and therefore reflecting either eventual selective responses or linkage disequilibrium with genes under divergent selection (Lewontin & Krakauer, 1973). In BayeScan, sample size was set to 10,000 and the thinning interval to 50. Loci with a posterior probability over 0.99, corresponding to a Bayes Factor > 2 (i.e., “decisive selection” (Foll & Gaggiotti, 2006)), were retained as outliers. In LOSITAN, a neutral distribution of *F*_ST_ with 1,000,000 iterations was simulated, with forced mean *F*_ST_ at a significance level of 0.05 under an infinite allele model for SNPs and under a stepwise model for microsatellites. To avoid pseudo replication, outlier analyses were conducted using a random sample of 300 individuals from each of the four genetic clusters identified with STRUCTURE (after excluding southern distant outgroups). Analyses were performed either using jointly the four sets of samples or using subsets, as appropriate.

Adaptation to local environments often occurs through natural selection acting on a large number of loci, each having a weak phenotypic effect. LFMM, “latent factor mixed model” (Frichot et al., 2013), was used to assess whether salinity or water temperature could be a potential selective pressure driving local adaptation by identifying loci showing unusual associations with these environmental factors compared to the genetic background. Thus, the environmental information used corresponded to the season of the year where fish are at its most temperature‐sensitive stages (Dahlke et al., 2020). This method, which has formerly proved to be efficient for a suite of scenarios of demographic history (Lotterhos & Whitlock, 2015; de Villemereuil et al., 2014), uses a linear mixed model to test for associations between genetic variation and environmental factors, while controlling for neutral genetic structure with (random) latent factors. Ten runs of LFMM were conducted using 1,000 sweeps for burn‐in and 10,000 additional sweeps. The number of latent factors was set at *K* = 4 according to STRUCTURE outcome as suggested by Frichot et al. (2013). The corresponding z‐scores of the ten replicates were combined following the recommendations described in Frichot and François (2015). First, the genomic inflation factor (*λ*) was obtained after computing the median of the squared (combined) z‐scores for each *K*, divided by the median of the chi‐square distribution with one degree of freedom. Finally, *p*‐values were adjusted using the genomic inflation factor (*λ*), and false discovery rates were set using the Benjamini and Hochberg (1995) algorithm.

In addition, the relationship between genetic distance (*F*_ST_) and each environmental factor was examined using Mantel (1967) tests to investigate whether the correlations conformed the expectations of “Isolation by Environment” (IBE); *that is,* pattern in which genetic differentiation increases with environmental differences irrespective of geographic distance (Wang & Bradburd, 2014), as opposed to “Isolation by Distance” (IBD), which refers to the increase of genetic differentiation with geographic distance as a result of restricted gene flow and drift (Rousset, 1997; Slatkin, 1993; Wright, 1943). Environmental distances to test for IBE were calculated as the Euclidean pairwise differences of the corresponding environmental factors. Mantel tests were conducted with a program called Pattern Analysis, Spatial Statistics and Geographic Exegesis, PASSaGE2 (Rosenberg & Anderson, 2011).

Allele frequency shifts at outlier loci are expected to be driven by selective responses toward strong ecological gradients leading to local adaptation, either due to directly associated genes or through hitchhiking (linkage) with associated genes (Gagnaire et al., 2015). Low‐frequency alleles can also reach high frequencies through allele surfing during population range expansion (Excoffier & Ray, 2008). Major allele frequencies (MAF) per sample were displayed through heatmaps and graphs as appropriate.

## 3 RESULTS

### 3.1 Summary statistics

Genetic diversity measured as observed and expected heterozygosity for individuals genotyped with SNPs showed low to intermediate values in Landvik, compared with relatively highest values found in all the Norwegian fjord samples, and the lowest estimates observed in the southern outgroups (Table 1). However, genetic diversity assessed as allelic richness, *H_o_* and *uH_e_* using microsatellites consistently displayed the lowest values in Landvik (Table 1).

### 3.2 Genetic differentiation

All the approaches used to compare Landvik with the reference samples highlighted the distinctness of the lake sprat, putting also in evidence the low gene flow occurring between the brackish lake and adjacent areas. Pairwise *F*_ST_ heatmaps for SNP‐genotyped samples (Table S1 in the Supplementary Information) revealed high and similar levels of differentiation between Landvik and the samples taken in the North Sea, Kattegat–Skagerrak, and the Baltic Sea (average *F*_ST_ 0.124), whereas the mean differentiation between Landvik and Norwegian coastal sprat, albeit high, was but nevertheless lower (0.080). However, Landvik samples did not display a homogeneous behavior as the lowest levels of differentiation were found against the most recent of the samples (LAND19), the only of the samples with sprat of age 1. The highest degree of divergence was found between Landvik and the outgroup samples (i.e., Mediterranean–Black Sea). Likewise, at microsatellites, the differentiation between Landvik and the Norwegian fjord samples (average *F*_ST_ of 0.095) was lower than versus the North Sea (*F*_ST_ = 0.117) and Baltic samples (*F*_ST_ = 0.137). In comparison, levels of differentiation within Norwegian fjord samples were very low (average *F*_ST_ of 0.003) despite the large geographic distances (Table A2 in Appendix 1).

**FIGURE 3:**
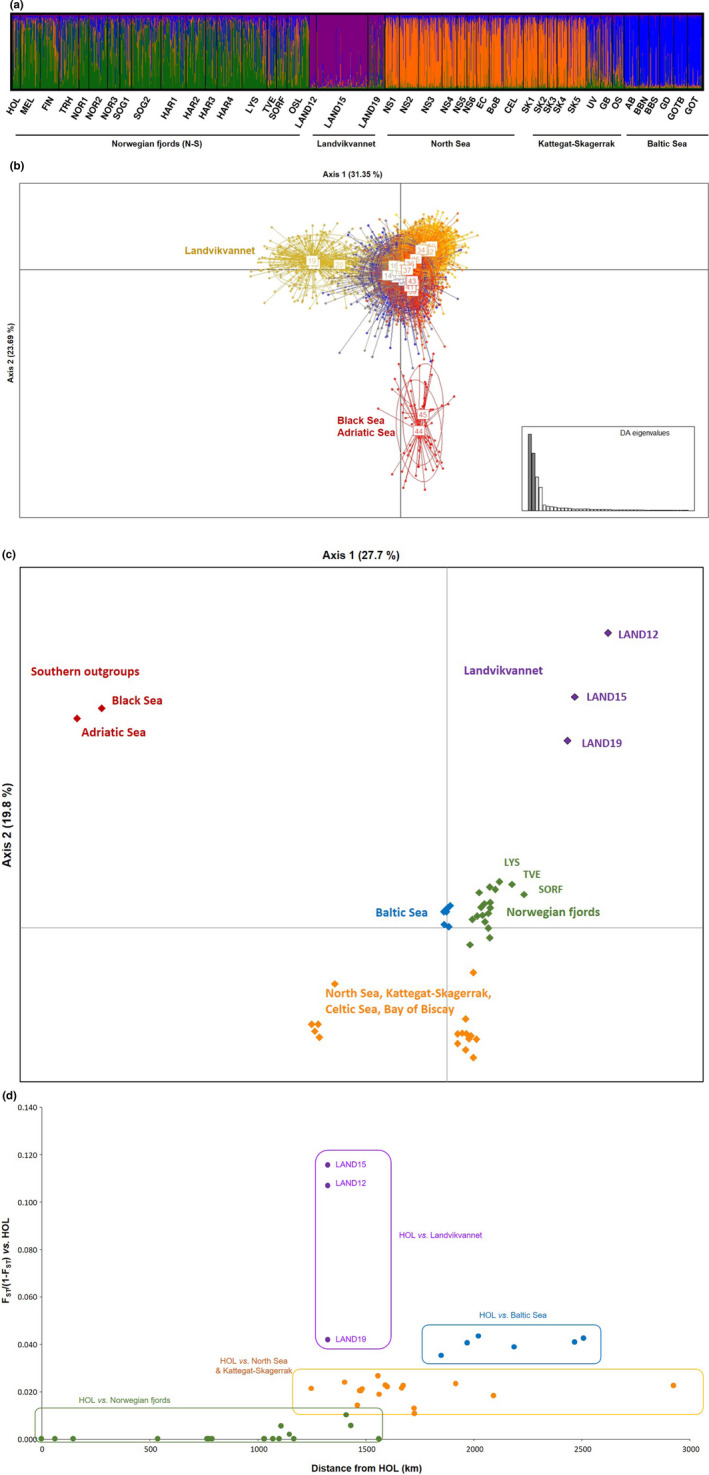
Relationship between Landvikvannet sprat and the reference samples genotyped at 91 SNP loci according to (a) STRUCTURE, (b) DAPC, and (c) PCoA. Plot in d) represents genetic distance measured as pairwise *F*_ST_/(1 − *F*_ST_) between the northernmost site (HOL) and each of the 41 remaining ones versus the corresponding shortest water distance (in km). Analyses in a) and d) were performed without the distant southern outgroups to increase the resolution

**FIGURE A1:**
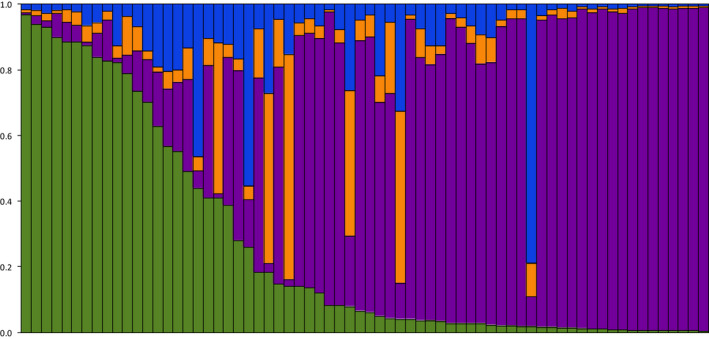
Extract of STRUCTURE barplot corresponding to LAND19 with individuals ordered by increasing ancestry to the Norwegian cluster (green). The rest of the clusters are depicted by the following colors: purple (Landvikvannet), blue (Baltic Sea) and orange (North Sea–Kattegat–Skagerrak)

**FIGURE A2:**
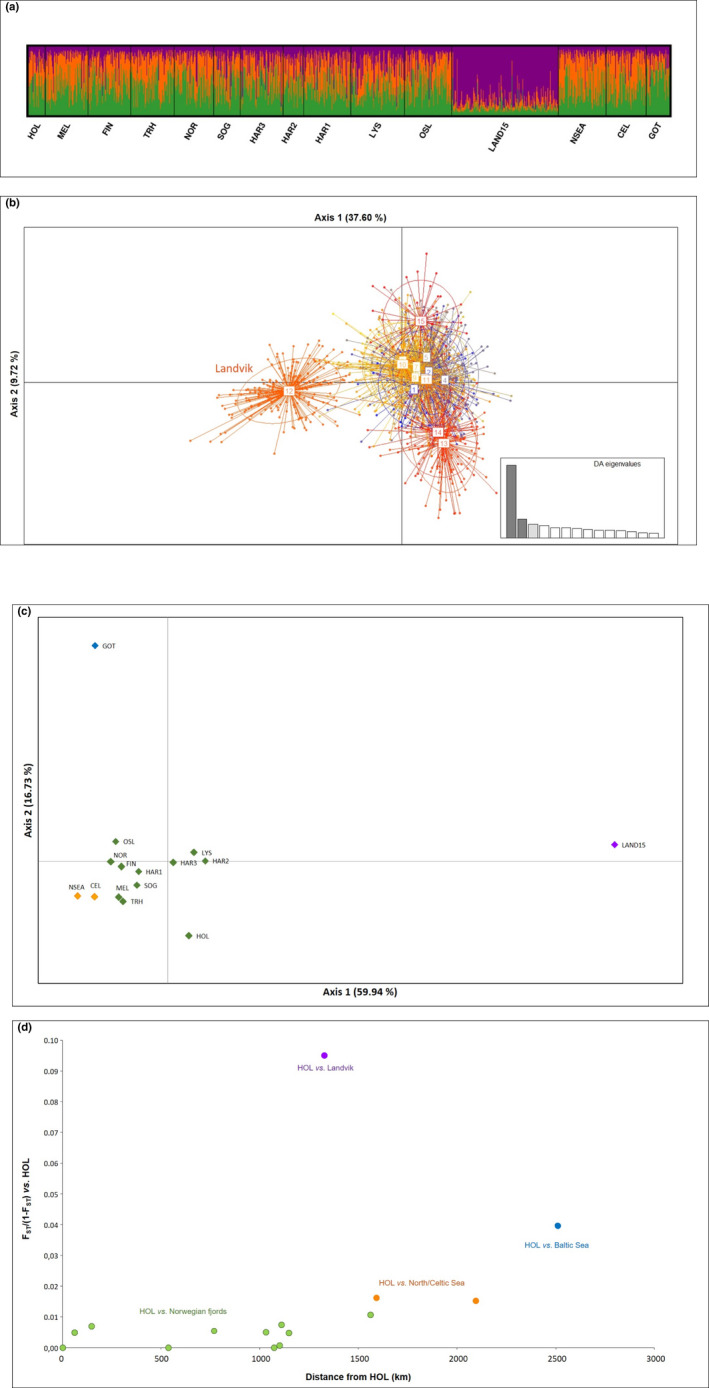
Relationship between Landvikvannet sprat and the 14 reference samples genotyped at microsatellites according to (a) STRUCTURE analysis, (b) DAPC, and (c) PCoA. The plot in (d) represents genetic distance measured as pairwise *F*_ST_/(1 − *F*_ST_) between the northernmost site (HOL) and each of the 14 remaining ones versus the corresponding shortest water distance (in km)

**FIGURE A3:**
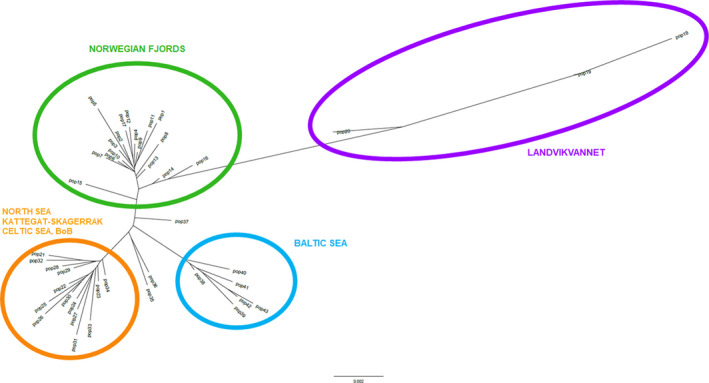
Origin of Landvikvannet sprat: Neighbor‐joining tree placing Landvikvannet in context with the 40 reference samples after removing the SNP loci candidates to directional selection flagged by LOSITAN and BayeScan. Names of sampling sites by numbers can be retrieved in Table 1

STRUCTURE was conducted without the southern samples from the Mediterranean and Black Sea in order to increase resolution on the target area of the study and its immediately surrounding seas. Evanno test identified a first hierarchical level of division at *K* = 2 that clustered Norwegian fjords and Landvik away from the remaining samples (see Table A3 in Appendix 1) whereas STRUCTURESelector identified four distinct clusters: (a) Norwegian fjords, (b) Landvik, (c) North Sea–Kattegat–Skagerrak, and (d) Baltic Sea (Figure 3a). The DAPC plot including all samples was built after retaining 80 principal components (PCs) and revealed three main groups: Landvik, the southern samples, and the remaining collections. Axis 1, explaining 31.4% of the variation, discriminated Landvik sprat from the bulk of the three main genetic clusters (Norwegian fjords, North Sea–Kattegat–Skagerrak, and Baltic Sea) with very little overlapping (Figure 3b). In agreement with estimates for pairwise *F*_ST_, this ordination on the first axis confirmed that the oldest samples from Landvik (LAND12, LAND15) were genetically more differentiated than the most recent sample LAND19. Axis 2, accounting for 23.7% of the variation, separated the southern outgroups. The DAPC highlighted that the level of differentiation between Landvik and geographically close samples was similar to the differentiation between Northern and Southern European sprat. Likewise, axis 1 in PCoA, explaining 27.7% of the variation, separated the southern outgroups, whereas axis 2 (19.8% variance explained) discriminated Landvik samples from the three remaining genetic clusters (Figure 3c). Plotting pairwise genetic distance between the northernmost sample (HOL) and each of the samples against the shortest water distance between the same pairs showed that the comparisons with Landvik strongly deviated from any geographically derived expectations (Figure 3d), particularly for the oldest samples (LAND12 and LAND15). Likewise, the correlation between the matrix of pairwise *F*_ST_ (without southern outgroups) and the matrix of Euclidean geographic distances did not conform with Isolation by Distance (*R_xy_* = 0.017, *p* = .359). Finally, GeneClass2 showed that across all samples 86% of the individuals genotyped at SNPs were correctly assigned to their respective clusters (Table A4 in Appendix 1). The correct self‐assignment per cluster ranged from 84% for Norwegian fjords to 100% in the Mediterranean Sea outgroups. In Landvik, 89% of the individuals were correctly assigned to the Landvik cluster, albeit with temporal differences: In 2012 and 2015, the percentage of correct assignment to cluster was of 96%–98%, respectively, whereas in 2019, it dropped to 60% as 21 individuals (i.e., 30% of the total in LAND19) were assigned to the Norwegian fjord cluster, 11 of them to the neighboring coastal samples (i.e., LYS, SORF, and TVE). As seven out of the 21 individuals showed an ancestry of *q* > 0.8 to the Norwegian fjord cluster, the hypothesis of them being migrants is plausible (see Figure A1 in Appendix 1). The 15 samples genotyped with microsatellites reproduced the patterns of genetic differentiation and clustering found with SNPs and clearly depicted the distinctness of Landvik sample (Figures A2, A3 in Appendix 1).

### 3.3 Genetic relationships of Landvik sprat

**FIGURE 4:**
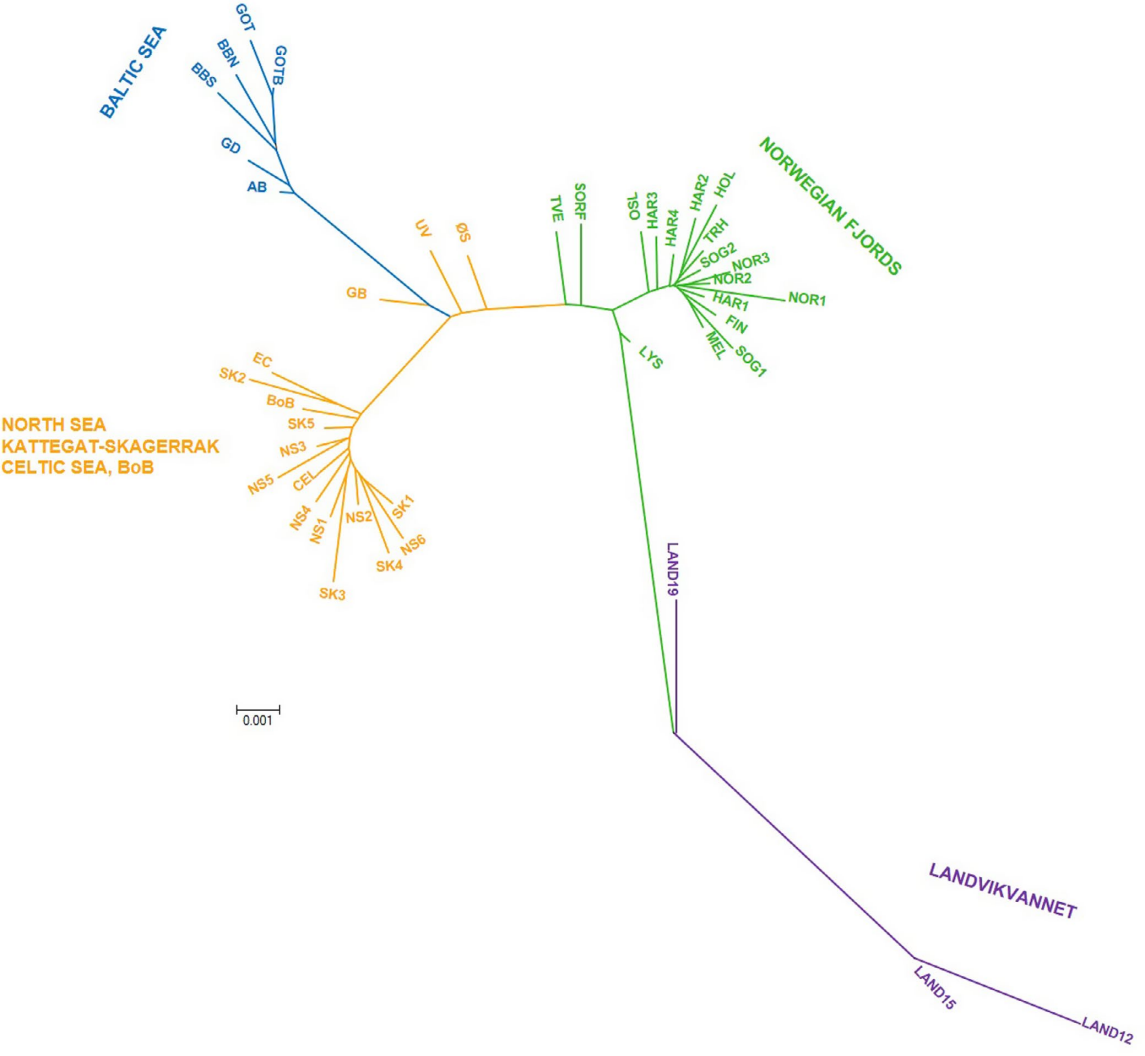
Origin of Landvik sprat: Neighbor‐joining tree placing Landvik in context with the reference samples genotyped at 91 SNP loci (NJ tree using after removing the loci under positive selection can be found in Figure A3 in Appendix 1). To increase the resolution, analyses were performed after excluding the distant southern outgroups

The determination of the origin of Landvik sprat is hampered by the high levels of differentiation between this population and the reference samples. Pairwise *F*_ST_ between Landvik and Norwegian fjord sprat were lower than any of the remaining comparisons hence revealing higher genetic relatedness than to brackish Baltic sprat (Table S1 in Supplementary File). Likewise, the Norwegian samples from LYS, TVE, and SORF, which are the geographically nearest to Landvik, were also the genetically closest (Figure 3c). Furthermore, the NJ tree built with all the SNPs not only highlighted the distinctness of Landvik, but also showed that the lake sprat could stem from the sprat of the Norwegian fjords as Landvik shared a node in the phylogenetic tree with LYS (Figure 4).

### 3.4 Selection tests and detection of loci associated with environmental factors

**FIGURE 5:**
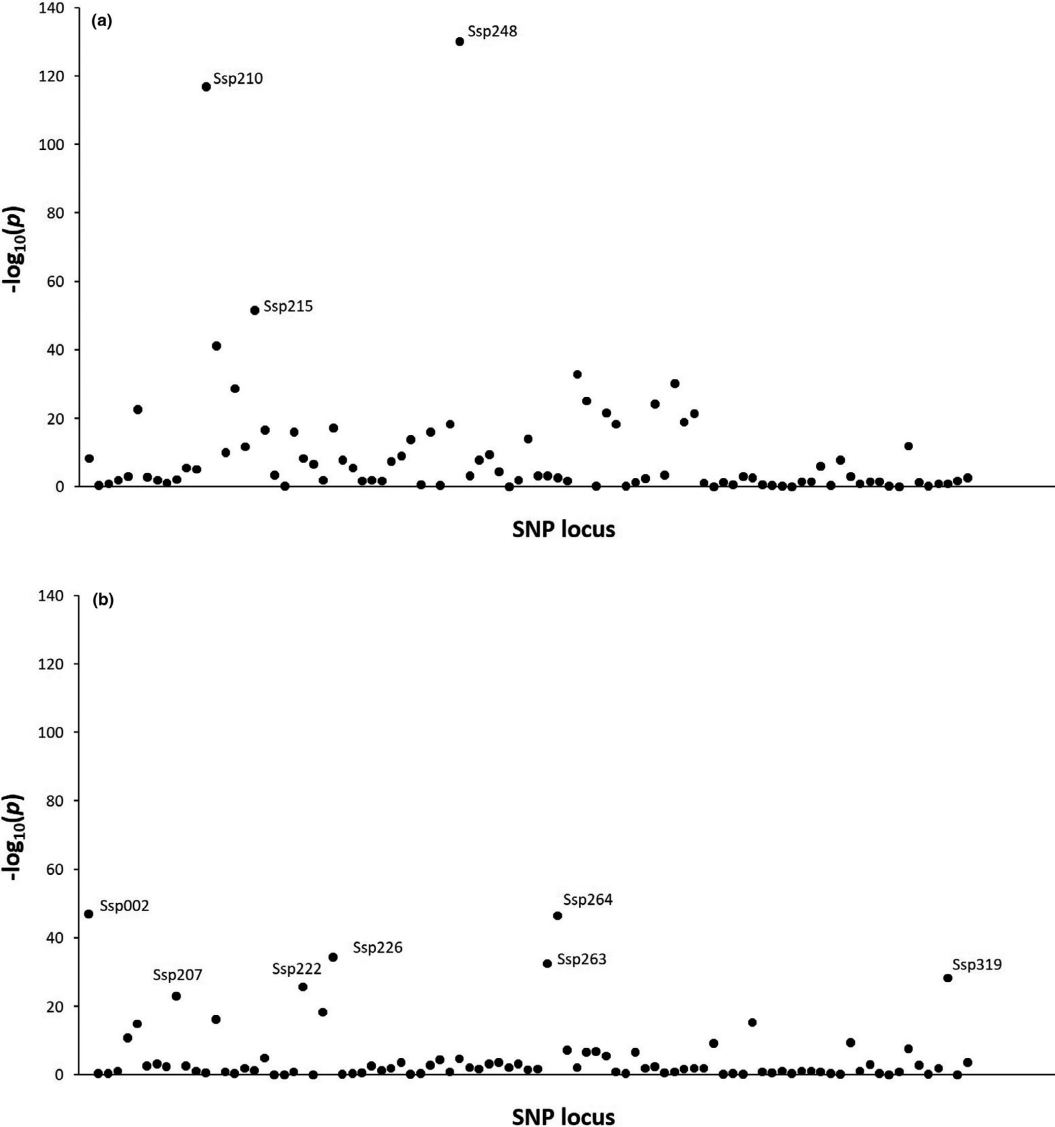
Manhattan plot from LFMM analysis for a) salinity and b) temperature, both measured during summer (average during the period 2005–2012) at 10 m of depth. Highlighted loci showed significant associations

Both outlier detection analyses (LOSITAN and BayeScan) as well as LFMM were conducted after excluding the southern distant groups due to their low sample size. LOSITAN reported four loci (4.4%) under positive selection, whereas BayeScan reported two, one of them in agreement with LOSITAN (Table 2). After genomic inflation correction, LFMM identified three loci associated with salinity and seven with temperature (Table 2), although the strength of the association was larger with salinity (Figure 5a,b). Locus Ssp263 was associated with temperature as well as flagged as an outlier by both procedures, whereas locus Ssp210 was associated with salinity, flagged as outlier with BayeScan and marginally with LOSITAN. Locus Ssp248, the one showing the strongest association with salinity (log_10_(P0) = 130.1), was annotated to the vicinity of a predicted protein kinase C epsilon in the herring genome whereas only one of the loci associated with temperature could be annotated, that is, locus Ssp319 to TOG array regulator of axonemal microtubules 1 also in the herring genome (Table 2). The relationships between genetic distance and environmental factors revealed that the correlation between the matrices of *F*_ST_ and salinity followed an Isolation by Environment pattern (*R*_xy_ = 0.47, *p* = .0001), conversely to the lack of correlation with temperature (*R*_xy_ = 0.05, *p* = .404).

**TABLE 2:** LFMM analysis for salinity and temperature (measured both in summer at 10 m depth)

Locus	Annotation	LFMM, log_10_(PO)		Global candidate loci		Pairwise candidate loci (LOSITAN)		
		Salinity	Temperature	LOSITAN	BayeScan	Norway vs. Landvik	Norway vs. Baltic	Landvik vs. Baltic
Ssp248	Protein kinase C epsilon	130.07	4.54	0.976	0.056	0.624	0.993	0.774
Ssp210		116.82	0.51	0.990	2.308	0.972	1	‐100
Ssp215	ATP‐dependent 6‐phosphofructokinase liver‐like	51.50	1.28	0.750	‐1.190	0.919	0.675	0.702
Ssp253		9.35	3.07	0.996	‐0.852	0.917	0.821	1
Ssp263		3.10	32.36	1	2.920	0.658	0.500	1
Ssp213	Tensin‐2‐like	28.52	0.41	1	0.502	0.997	0.500	1
Ssp268		25.08	6.50	1	0.419	0.998	0.765	1
Ssp279		30.16	0.83	0.799	‐1.192	1	0.745	0.250
Ssp260		13.95	1.48	0.991	‐1.060	0.994	0.493	0.999
Ssp315		11.83	7.58	0.267	‐1.020	1	0.500	0.500
Ssp290		2.55	15.14	0.374	‐0.987	1	0.050	‐100
Ssp272	Neurochondrin transcript variant X1‐3	18.18	0.80	0.606	‐1.226	0.465	1	0.500
Ssp226		17.21	34.26	0.720	0.001	0.437	1	0.500
Ssp269		0.12	6.80	0.621	‐1.173	0.103	1	0.002
Ssp286		0.05	9.14	0.435	‐1.182	0.414	1	0.500
Ssp302	Procollagen C‐endopeptidase enhancer 2‐like	7.87	0.18	0.638	‐1.110	0	1	1
Ssp225		1.77	18.25	0.835	0.040	0.232	1	1
Ssp300	Zinc finger protein 184‐like transcript variant	5.88	0.75	0.468	‐1.211	0.500	0.648	1
Ssp319	TOG array regulator of axonemal microtubules 1	0.82	28.17	0.972	0.454	0.343	0.826	1
Ssp222		8.26	25.54	0.500	‐0.602	0.269	0.500	‐100
Ssp002		8.19	46.96	0.800	‐1.033	0.944	0.684	0.830
Ssp264		2.57	46.41	0.884	‐0.615	0.281	0.925	0.807
Ssp207		2.10	22.86	0.722	‐1.195	0.851	0.383	0.854

Note

Outlier analyses were performed using 300 randomly sampled individuals per genetic cluster instead of geographically explicit samples. Cells shaded in dark grey depict significant associations at LFMM after genomic inflation correction as well as candidates for positive selection according to LOSITAN after FDR correction (P(Simul F_ST_<sample F_ST_)) and BayeScan (log_10_(PO)). Cells shaded in light grey depict candidates to balancing selection. BayeScan did not detect deviations from neutrality in the pairwise comparisons. Flanking sequences of SNP loci were blasted against the GenBank and annotated genes in the vicinity of SNP markers were indicated as appropriate (empty cells depict no hit). All the annotated genes are Predicted for *Clupea harengus*.

Furthermore, outlier detection analyses were conducted for the same clusters of 300 randomly sampled individuals albeit in a pairwise fashion through comparisons involving the Norwegian sprat, Landvik, and the Baltic Sea (Table 2). Locus Ssp210, showing strong association with salinity, was reported to be a candidate to positive selection in the comparison between marine and brackish samples in Norway versus Baltic and marginally in Norway versus Landvik, whereas no positive selection was reported between brackish environments. On the other side, four loci (Ssp253, Ssp263, Ssp300, and Ssp319) were candidates to directional selection in the comparison between brackish environments (Landvik vs. Baltic Sea) but not in the comparison's marine versus brackish, which could eventually support rejecting the hypothesis of the origin of the lake sprat being in the Baltic Sea. Major allele frequency per sample for the loci showing the strongest association with salinity was assessed in samples from contrasting environments (Table 3) and revealed a similar pattern in low salinity waters (i.e., Landvik and the Baltic Sea) as opposed to marine waters (Norwegian fjords), which could suggest that Landvik sprat evolved from the Norwegian make‐up to adapt to low salinity environments. Conversely, no temperature‐related pattern for a similar process was obvious.

**TABLE 3:** Heatmap of Major Allele Frequency per sample for the loci showing the strongest association with salinity and temperature according to LFMM analyses

	Sample no.	Sample	Salinity			Temperature		
			Ssp210	Ssp215	Ssp248	Ssp264	Ssp002	Ssp226
Norwegian fjords	1	HOL	0.767	0.758	0.871	0.767	0.629	0.850
	2	MEL	0.679	0.705	0.794	0.753	0.625	0.763
	3	FIN	0.653	0.653	0.793	0.703	0.643	0.800
	4	TRH	0.813	0.728	0.812	0.763	0.595	0.835
	5	NOR1	0.756	0.635	0.782	0.689	0.577	0.821
	6	NOR2	0.791	0.682	0.740	0.703	0.562	0.778
	7	NOR3	0.792	0.744	0.786	0.727	0.551	0.765
	8	SOG1	0.630	0.804	0.755	0.707	0.723	0.809
	9	SOG2	0.806	0.717	0.763	0.719	0.586	0.809
	10	HAR1	0.745	0.702	0.825	0.747	0.600	0.828
	11	HAR2	0.727	0.662	0.767	0.799	0.500	0.786
	12	HAR3	0.830	0.630	0.750	0.678	0.576	0.784
	13	HAR4	0.755	0.737	0.824	0.712	0.590	0.806
	14	LYS	0.788	0.670	0.726	0.652	0.621	0.840
	15	TVE	0.824	0.614	0.730	0.703	0.586	0.878
	16	SORF	0.869	0.588	0.842	0.738	0.700	0.893
	17	OSL	0.843	0.684	0.831	0.787	0.642	0.801
Landvik	18	LAND12	1.000	0.963	0.595	0.685	0.788	0.846
	19	LAND15	0.988	0.924	0.653	0.706	0.909	0.955
	20	LAND19	0.919	0.836	0.688	0.694	0.784	0.904
Baltic Sea	38	AB	0.992	0.741	0.518	0.941	0.698	0.983
	39	BBN	0.987	0.859	0.472	0.936	0.697	1.000
	40	BBS	1.000	0.780	0.563	0.940	0.725	1.000
	41	GD	0.991	0.857	0.580	0.945	0.688	0.982
	42	GOTB	0.981	0.849	0.500	0.955	0.673	1.000
	43	GOT	0.991	0.858	0.434	0.971	0.625	1.000

**FIGURE 6:**
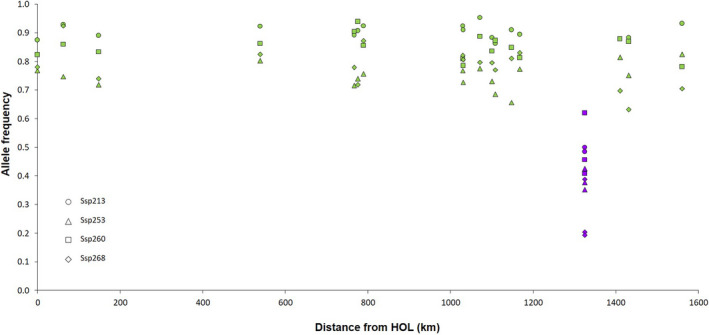
Major allele frequency for the four loci showing the largest differentiation in Landvikvannet compared to the Norwegian sprat. Allele frequency per sample was plotted versus the shortest water distance between each site and HOL (the northernmost one). The coloring pattern followed STRUCTURE barplot, *that is,* green for the Norwegian fjords and purple for Landvikvannet

Finally, the major allele frequency of seven of the SNPs (Ssp253, Ssp321, Ssp260, Ssp268, Ssp213, Ssp251, and Ssp236) showed a remarkable drop in Landvik compared with the Norwegian fjord samples illustrating a change that could have happened in less than 132 years (see Figure 6 for four of them).

## 4 DISCUSSION

The brackish lake Landvik, created after excavating a 3 km long canal to the sea in 1880, represents a model system in which to investigate the potential for marine organisms to adapt to rapidly emerging new environments in the marine realm. Here, we showed that European sprat, a small pelagic marine fish, were able to colonize and develop a genetically highly distinct population in few decades. The level of differentiation observed between samples from Landvik and all other reference samples of sprat was equivalent to the genetic differentiation displayed among the most geographically distant populations of sprat. This level of differentiation has been achieved in a maximum of 132 years, as computed from the completion of the canal until the sampling date of LAND12, which would mean some 65 generations of sprat. Thus, Landvik adds to the three distinctive genetic clusters formerly described in European sprat, that is, Norwegian fjords, North Sea–Kattegat–Skagerrak, and Baltic Sea (Quintela et al., 2020). The study also suggests signatures of contemporary adaptation to brackish habitat in Landvik sprat population, which represents a potential model system to study parallel evolution in comparison with the Baltic.

### 4.1 Origin of the Landvik population

The relationship between genetic differentiation and shortest water distance revealed that samples from Landvik strongly departed from any geographically driven expectation (see Figure 3d), a situation also described for three spine sticklebacks (*Gasterosteus aculeatus* Linnaeus, 1758), where populations inhabiting anthropogenic modified habitats deviated from the general pattern of Isolation by Distance (Scharsack et al., 2012). Another striking characteristic of the Landvik sprat population is the relatively low genetic diversity displayed by microsatellite markers in terms of *H_o_*, *uH_e_*, and allelic richness. The only sample that exhibited a comparably low allelic richness was collected in Gotland (Baltic Sea), in the brink of Baltic sprat's spawning habitat where salinity conditions approach to those impeding larval survival (Sjöblom & Parmanne, 1980). Landvik, thus, adheres to the pattern previously described in the Baltic, where the combination of young age, extreme conditions, and limited habitat size leads Baltic populations to often have less intraspecific genetic diversity than their counterparts in the open Northeast Atlantic (Johannesson & Andre, 2006).

Landvik is also inhabited by a taxonomically close species to sprat: the Atlantic herring. Landvik herring are considered as a self‐sustaining and somewhat stationary population, characterized by slower growth, smaller length at maturity, lower vertebral count, shorter life span, higher relative fecundity, and divergent genetic profiles compared to the migratory oceanic herring in other parts of the Norwegian waters (Eggers, 2013; Eggers et al., 2014; Silva et al., 2013). Meristic trait vertebral count is often used as a population identifier in herring (e.g., Berg et al., 2017; Mosegaard & Madsen, 1996; Rosenberg & Palmén, 1981), and the observation that vertebral count in Landvik herring is similar to that in herring populations in the brackish Western Baltic Sea has led to the hypothesis that Landvik was colonized by low salinity adapted herring of Western Baltic Sea origin (Berg et al., 2019; Eggers et al., 2014). In addition, factorial crossing experiments performed at a range of salinities ranging from 6 to 35 revealed adaptation of herring populations to their native salinity conditions and also that adaption to salinity is transmitted to the offspring within the following generation (Berg et al., 2019). In contrast to herring, which rely on a benthic spawning habitat for depositing eggs, sprat is a pelagic spawner. As such, salinity may exert an even stronger selection pressure in sprat to avoid neutrally buoyant eggs from sinking into deeper anoxic water layers, as has for example been observed in Atlantic cod, *Gadus morhua*, adapted to spawning in brackish waters (Berg et al., 2015; Nissling et al., 1994). In both cod and herring, local adaptation is implied to be swift and ongoing, and working on standing genetic variation (e.g., Berg et al., 2015; Lamichhaney et al., 2012).

The origin of Landvik sprat is unknown, but, based on inference from herring, it would be conceivable that the lake could have also been colonized by fish from the Baltic Sea, already adapted to brackish waters, given the parallelism in the environmental conditions. However, the analysis of Landvik in conjunction with the reference samples available in this study does not appear to support the hypothesis of the Baltic Sea as the source, but points toward founders from Norwegian fjordic sprat. This is particularly endorsed by the lower genetic differentiation between Norwegian sprat and Landvik, as well as by the neighbor‐joining tree showing that Landvik sprat stems from the Norwegian cluster. Taking into consideration that NJ analyses are sensitive to outliers, the tree was recalculated after purging the candidate loci to positive selection detected by LOSITAN and BayeScan. The new NJ tree confirmed that the node from which Landvik sprat stem was the Norwegian sample, LYS (see Figure A3 in Appendix 1). Furthermore, Landvik sprat displays a suite of features that concur with founder effects such as strongly drifted allele frequencies at both microsatellites and SNPs, together with low genetic diversity at microsatellites assessed as allelic richness, *H_o_* and *uH_e_* in comparison with the Norwegian samples. Similarly, losses of genetic diversity, a signature compatible with historical founder effect, have been reported in other fish species in the face of anthropic challenges: *for example,* Mango tilapia *Sarotherodon galilaeus* (Linnaeus, 1758) in the Sea of Galilee (Borovski et al., 2018), American paddlefish *Polyodon spathula* (Walbaum in Artedi 1792) stocked in Poland from United States (Kaczmarczyk et al., 2012), or the introduced grass carp *Ctenopharyngodon idella* (Valenciennes in Cuvier & Valenciennes, 1844) with respect to its native Chinese ranges (Chen et al., 2012). Likewise, the isolation of the live‐bearing fish Caterina allocota *Allotoca catarinae* (de Buen, 1942) from another species of the same complex dated ~1900 years ago represents the first evidence of fish species translocation by a pre‐Hispanic culture of Mexico (Corona‐Santiago et al., 2015). Finally, the sample taken in Landvik in 2019 included 21 individuals (31%) with a genetic profile compatible with being migrants from surrounding Norwegian fjords as denoted by an inferred ancestry to the Norwegian STRUCTURE cluster of *q* > 0.8 and by being assigned to this cluster by GeneClass. However, only one individual approached this level of ancestry for the Baltic Sea cluster. The fact of finding potential migrants coming from the coastal Norway, but not from the Baltic Sea, would further support the hypothesis of the Norwegian fjords as a source of Landvik sprat. Despite these levels of gene flow, outlier loci revealed patterns of population structure that support postsettlement selection and suggest that strong selective forces could be acting and therefore causing local adaptation.

### 4.2 Adaptation as a consequence of brackish water colonization

Transitions from marine to freshwater habitats constitute dramatic shifts between adaptive habitats that have occurred not only on macroevolutionary time scales, but also in the recent past (Lee & Bell, 1999). During the last two centuries, humans have been changing connections between freshwater and marine ecosystems thus facilitating freshwater introductions (Crook et al., 2015). Drastic differences in salinity, parasites, competitors, and predators between marine and freshwater environments exert divergent selective pressures on the corresponding populations. Salinity showed strong associations with 3.3% of the loci analyzed in the present study. The genetic change experienced by the Norwegian sprat colonizing Landvik could be attributed to the strong directional selection driven by the low salinity in the lake. Rapid evolutionary changes are predicted in the face of strong selection following habitat shifts or environmental disturbances (Burke & Long, 2012; Kopp & Matuszewski, 2014; Losos et al., 1997; Turcotte et al., 2011), as happened in Landvik when the lake was artificially connected to the sea circa 150 years ago. Similar processes have been documented in other species such as the threespine stickleback, which managed to evolve from oceanic ancestors to colonize the freshwater ponds that were formed during uplift caused by the Great Alaska Earthquake in 1964 (Lescak et al., 2015). Adaptation of a newly established resident population to the brackish environment often proceeds very fast, over the course of several decades (Barrett et al., 2008; Lescak et al., 2015; Marques et al., 2018). Data on adaptation associated with salinity have been reported in fish moving from high to low salinity such as sticklebacks (DeFaveri & Merilä, 2014; McCairns & Bernatchez, 2010), Atlantic killifish *Fundulus heteroclitus* (Linnaeus, 1766) (Whitehead et al., 2011), and alewives *Alosa pseudoharengus* (A. Wilson, 1811) (Velotta et al., 2014).

Outlier loci experiencing adaptive selection based on environmental conditions have also been described in other Clupeids. Ruggeri et al. (2016) related population divergence in microsatellite outlier loci in relation to salinity, oxygenation, and temperature in the European anchovy *Engraulis encrasicolus* (Linnaeus, 1758) in the Adriatic Sea. For the same species, Catanese et al. (2017) reported that the selective pressure related to river mouths acts on the same genes in distant areas in the Atlantic Ocean, Tyrrhenian, and North Adriatic Sea. These SNP outliers were also associated with salinity variability or involved in a critical stage of fertilization process.

The Baltic Sea was formed after the latest ice age, approximately 10,000–15,000 years ago, although its “ecological age” is circa 8,000 years (Lass & Matthäus, 2008). The combination of young geological age and contrasting environmental conditions to the surrounding oceans resulted in fast processes of adaptive evolution, which led to species living in the edge of their physiological tolerance (Ojaveer et al., 2010). The degree of differentiation between marine and brackish sprat is higher in the comparisons Norway fjords versus Landvik (mean *F*_ST_ = 0.080, range 0.029–0.117) than in Norway fjords versus Baltic (mean *F*_ST_ = 0.037, range 0.026–0.047). Hypothesizing that the origin of Landvik sprat is the Norwegian fjord sprat invokes the possibility that the genetic changes occurring in the lake took place in a maximum of 65 generations (<132 years) rather than gradually over thousands of years. This hypothesis was also put forward in the threespine stickleback, which achieved in 50 years similar levels of divergence as populations that had diverged thousands of years ago (Lescak et al., 2015). Most likely, such rapid adaptation to a new environment cannot depend on de novo mutations and must rely primarily on standing genetic variation (Matuszewski et al., 2015; Dolph Schluter & Conte, 2009) as it has been demonstrated in the threespine stickleback populations (Terekhanova et al., 2019). The high speed of adaptation of Landvik population to the brackish environment probably has been possible by freshwater tolerance alleles being present in ancestral marine populations.

### 4.3 Parallel or convergent evolution under similar selection pressures?

Adaptation to a radically different environment is likely to be genetically complex and to involve many loci, as it has been shown for other species (Terekhanova et al., 2019). Locus Ssp210, showing strong association with salinity, was reported to be a candidate to positive selection in the comparison between marine and brackish samples (in Norway vs. Baltic and marginally in the comparison Norway vs. Landvik) but not in the comparison between brackish environments (Lanvik vs. Baltic), which could suggest parallel or convergent evolution processes in Landvik and the Baltic Sea diverging from marine sprat. Despite the geographic proximity between Landvik and the Norwegian coastal sites, strong genetic divergence is found among those samples, probably due to differences in abiotic parameters (salinity) between habitats.

Parallel evolution under similar selection pressure has been widely observed in populations of the same species, *for example,* in bacterial experiments (Baym et al., 2016), recurrent adaptations of pathogens to their hosts (Collins & Didelot, 2018), and marine threespine sticklebacks that have independently colonized many freshwater habitats (Stuart et al., 2017). Baltic Sea and Landvik sprat populations could well be the results of parallel or convergent evolution (Arendt & Reznick, 2008), as it has been observed in lake and stream sticklebacks (Colosimo et al., 2005; Stuart et al., 2017). They dwell in discrete and divergent habitats, and are derived from ancestral marine populations, increasing the likelihood of them reusing similar ancestral genetic variants for adaptation. However, unlike the case of some stickleback populations that colonized lakes and streams after the last glaciation from the same ancestral population (Bolnick et al., 2018; Therkildsen et al., 2019), the history of Landvik and the Baltic Sea are different and could lead to a nonparallel evolution.

Despite the limitations of the current set of SNPs markers, the candidate outliers that distinguish derived brackish populations from the ancestral marine populations are not same between Landvik and Baltic Sea populations, thus not showing a pattern of molecular parallelism, contrary of what has been detected in sticklebacks colonizing different lakes (Terekhanova et al., 2014). Marine populations of stickleback harbor, at low frequencies, alleles that confer adaptation to freshwater (Schluter & Conte, 2009), presumably due to the gene flow from coastal freshwater populations (Bassham et al., 2018). This population structure and history would provide many opportunities for parallel evolution when new freshwater populations were established from the marine stickleback population (Stern, 2013). In sticklebacks, that genetic parallelism is seen on finer geographic scales (Jones et al., 2012; Nelson & Cresko, 2018) but not globally, a pattern attributed to founder events and the loss of genetic diversity following colonization of the Atlantic (Fang et al., 2020). Landvik adaptation to brackish waters could have followed a similar pattern, where adaptation independent from Baltic populations has been a consequence of demographic forces of the founder event of the lake from the Norwegian coast populations. However, our study has strong limitations to disentangle whether it is a case of molecular parallelism or independent adaptation. Future genomic studies may help reveal the evolutionary history of the sprat and the molecular mechanisms involved in its different adaptations to brackish environments. Study the genetics of convergence can help shed light on fundamental questions in evolutionary biology, including whether natural selection is constrained and repeatable or instead characterized by many molecular paths to similar phenotypes.

The uniqueness of Landvik sprat suggests that an appropriate management should be considered for this population. A next step using whole‐genome sequencing will allow to further explore intraclusters standing genetic variation as well as the origin of Landvik population. Parallel evolution in response to similar environmental pressures strongly suggests evolution by natural selection; however, the underlying genetic basis of this process is unclear. Landvik sprat thus provides an excellent opportunity for testing the genomic aspects of evolutionary repeatability.

## CONFLICT OF INTEREST

None declared.

## AUTHOR CONTRIBUTIONS

**María Quintela:** Conceptualization (equal); formal analysis (lead); methodology (supporting); writing–original draft (lead); writing–review and editing (lead). **Àlex Richter‐Boix:** Formal analysis (equal); writing–original draft (lead); writing–review and editing (equal). **Dorte Bekkevold:** Conceptualization (equal); data curation (lead); formal analysis (supporting); funding acquisition (supporting); methodology (supporting); writing–original draft (supporting); writing–review and editing (supporting). **Cecilie Kvamme:** Conceptualization (equal); data curation (lead); funding acquisition (lead); project administration (lead); writing–original draft (supporting); writing–review and editing (supporting). **Florian Berg:** Data curation (equal); writing–original draft (supporting); writing–review and editing (supporting). **Eeva Jansson:** Data curation (equal); formal analysis (supporting); methodology (supporting); writing–original draft (supporting); writing–review and editing (supporting). **Geir Dahle:** Data curation (equal); methodology (equal); writing–original draft (supporting); writing–review and editing (supporting). **François Besnier:** Formal analysis (equal); methodology (equal); writing–original draft (supporting); writing–review and editing (supporting). **Richard D. M. Nash:** Data curation (supporting); writing–original draft (supporting); writing–review and editing (supporting). **Kevin A. Glover:** Conceptualization (equal); data curation (supporting); formal analysis (supporting); funding acquisition (equal); project administration (equal); writing–original draft (supporting); writing–review and editing (supporting).

## Supporting information

Supplementary Material

Table S1

## Data Availability

The raw data for SNPs and microsatellites are available in https://doi.org/10.5061/dryad.zs7h44j7h.

## APPENDIX 1

**TABLE A1:** Sprat genotyped at 8 microsatellites

Sample	*N*	No alleles	Ar	*H_o_*	*uH_e_*	*F*_IS_	Dev HWE (B)	Dev LD (B)
HOL	31	130	16.3	0.88 ± 0.03	0.87 ± 0.03	−0.04 ± 0.04	2 (0)	1 (0)
MEL	79	189	17.2	0.80 ± 0.05	0.88 ± 0.03	0.09 ± 0.04	5 (4)	2 (1)
FIN	79	185	16.7	0.80 ± 0.05	0.88 ± 0.03	0.09 ± 0.04	4 (3)	1 (1)
TRH	80	193	17.3	0.80 ± 0.05	0.88 ± 0.03	0.09 ± 0.04	3 (0)	1 (1)
NOR	73	182	16.7	0.81 ± 0.05	0.87 ± 0.03	0.07 ± 0.04	7 (5)	0 (0)
SOG	49	150	16.3	0.81 ± 0.05	0.89 ± 0.02	0.08 ± 0.05	2 (1)	3 (2)
HAR1	79	173	16.2	0.78 ± 0.04	0.87 ± 0.02	0.10 ± 0.04	4 (4)	0 (0)
HAR2	38	129	15.4	0.79 ± 0.02	0.85 ± 0.03	0.05 ± 0.03	2 (1)	1 (1)
HAR3	87	193	16.9	0.81 ± 0.03	0.87 ± 0.03	0.06 ± 0.03	4 (4)	0 (0)
LYS	99	195	16.5	0.80 ± 0.05	0.87 ± 0.02	0.08 ± 0.04	5 (4)	1 (1)
OSL	87	190	16.9	0.81 ± 0.04	0.87 ± 0.03	0.07 ± 0.03	4 (3)	0 (0)
LAND15	97	182	12.2	0.63 ± 0.08	0.70 ± 0.08	0.08 ± 0.06	5 (5)	0 (0)
NSEA	88	207	18.3	0.79 ± 0.05	0.89 ± 0.03	0.11 ± 0.04	6 (4)	0 (0)
CEL	74	193	17.9	0.83 ± 0.06	0.88 ± 0.03	0.06 ± 0.04	2 (1)	1 (1)
GOT	43	121	13.5	0.84 ± 0.04	0.82 ± 0.04	−0.04 ± 0.03	3 (0)	1 (0)

Note

Summary statistics per sampling site: Number of individuals; number of alleles; allelic richness (Ar); observed heterozygosity, *H_o_* (mean ± *SE*); unbiased expected heterozygosity, *uH_e_* (mean ± *SE*); inbreeding coefficient, *F*_IS_ (mean ± *SE*); number of deviations from Hardy–Weinberg equilibrium (HWE) at *α* = 0.05; number of deviations from Linkage Disequilibrium (LD) at *α* = 0.05 both before and after (B) Bonferroni correction.

**TABLE A2:** Sprat genotyped at 8 microsatellites

	HOL	MEL	FIN	TRH	NOR	SOG	HAR1	HAR2	HAR3	LYS	OSL	LAND15	NSEA	CEL	GOT
HOL	*	**0.040**	**0.009**	0.577	**0.021**	**0.047**	0.396	0.817	**0.020**	**0.005**	**0.001**	**0.000**	**0.000**	**0.000**	**0.000**
MEL	0.005	*	0.226	0.482	0.426	0.126	0.443	0.155	0.353	**0.000**	**0.002**	**0.000**	**0.000**	**0.000**	**0.000**
FIN	0.007	0.001	*	0.561	0.470	0.538	0.527	0.462	0.219	**0.000**	**0.012**	**0.000**	**0.000**	**0.000**	**0.000**
TRH	0.000	0.000	0.000	*	0.288	0.190	0.569	0.794	0.352	**0.000**	0.107	**0.000**	**0.000**	**0.000**	**0.000**
NOR	0.005	0.000	0.000	0.001	*	0.114	0.482	0.096	0.411	**0.006**	**0.001**	**0.000**	**0.000**	**0.000**	**0.000**
SOG	0.005	0.003	0.000	0.002	0.003	*	0.640	0.778	0.051	**0.044**	**0.007**	**0.000**	**0.001**	**0.000**	**0.000**
HAR1	0.001	0.000	0.000	0.000	0.000	0.000	*	0.997	0.331	0.462	0.065	**0.000**	**0.000**	**0.000**	**0.000**
HAR2	0.000	0.003	0.000	0.000	0.003	0.000	0.000	*	0.539	0.127	0.144	**0.000**	**0.000**	**0.000**	**0.000**
HAR3	0.005	0.001	0.001	0.001	0.000	0.003	0.001	0.000	*	**0.000**	**0.006**	**0.000**	**0.000**	**0.000**	**0.000**
LYS	0.008	0.007	0.006	0.007	0.004	0.003	0.000	0.003	0.005	*	**0.000**	**0.000**	**0.000**	**0.000**	**0.000**
OSL	0.011	0.005	0.004	0.002	0.006	0.006	0.002	0.002	0.004	0.011	*	**0.000**	**0.000**	**0.000**	**0.000**
LAND15	0.087	0.103	0.102	0.100	0.107	0.101	0.086	0.080	0.095	0.079	0.105	*	**0.000**	**0.000**	**0.000**
NSEA	0.016	0.007	0.007	0.008	0.010	0.008	0.011	0.014	0.009	0.015	0.010	0.117	*	0.163	**0.000**
CEL	0.015	0.008	0.008	0.007	0.010	0.012	0.011	0.019	0.009	0.017	0.011	0.114	0.002	*	**0.000**
GOT	0.038	0.025	0.020	0.025	0.020	0.026	0.023	0.032	0.023	0.024	0.019	0.137	0.027	0.030	*

Note

Heatmap of pairwise *F*_ST_ below diagonal and the corresponding *p*‐values after 10,000 permutations above diagonal. Cells in boldface type depict values significantly different from zero and the ones highlighted in pink depict values that retained significance after sequential Bonferroni correction.

**TABLE A3:** Sprat genotyped at 91 SNPs: Outcome of STRUCTURESelector and Evanno test after ten STRUCTURE runs from K1 to K7

K	Runs	STRUCTURESelector				Evanno test				
		MedMed	MedMean	MaxMed	MaxMean	Mean LnP(K)	Stdev LnP(K)	Ln′(K)	|Ln″(K)|	ΔK
1	10	1	1	1	1	−198719.85	0.05	NA	NA	NA
2	10	2	2	2	2	−195809.77	2.74	2,910.08	731.26	267.02
3	10	3	3	3	3	−193630.95	16.86	2,178.82	1,170.00	69.41
4	10	4	4	4	4	−192622.13	27.26	1,008.82	29.74	1.09
5	10	5	3	5	4	−191643.05	193.65	979.08	445.99	2.30
6	10	3	3	3	3	−191109.96	43.81	533.09	182.68	4.17
7	10	3	3	3	3	−190759.55	24.42	350.41	NA	NA
		**MedMedK**	**MedMeaK**	**MaxMedK**	**MaxMeaK**					
		**5**	**4**	**5**	**4**					

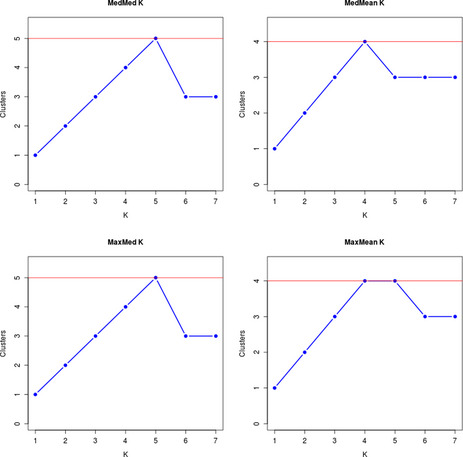
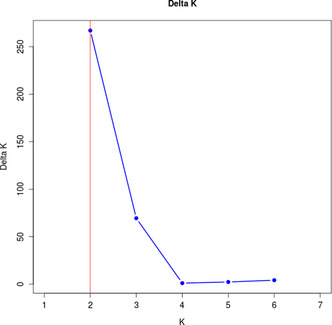

**TABLE A4:** Assignment analysis for individuals genotyped at SNPs to the different genetic clusters

	Norway	Landvik	NorthSea, Katt‐Skag	Baltic	Outgr	Correct self‐assign (%)
Norway	**987**	21	135	37	0	**83.6**
Landvik	23	**268**	7	2	0	**89.3**
NorthSea, Kattegat–Skagerrak	70	3	**808**	69	0	**85.1**
Baltic	10	0	18	**280**	0	**90.9**
Outgroups	0	0	0	0	**66**	**100.0**

Note

Values in the diagonal gray cells depict the correct self‐assignment to cluster (in numbers). The last column shows the same values but in percentage. Overall correct self‐assignment was 86%.
